# ATP6AP1 drives pyroptosis-mediated immune evasion in hepatocellular carcinoma: a machine learning-guided therapeutic target

**DOI:** 10.1007/s12672-025-02426-1

**Published:** 2025-04-25

**Authors:** Lei Tang, Xiyue Wang, Zhengzheng Xia, Jiayu Yan, Shanshan Lin

**Affiliations:** 1 Department of Infectious Diseases, The Second Affiliated Hospital of Anhui Medical University, Hefei, 230601 China; 2 The First Clinical College, Guangdong Medical University, Zhanjiang, 524023 Guangdong China

**Keywords:** ATP6AP1, Hepatocellular carcinoma (HCC), Pyroptosis, Tumor microenvironment (TME), Resting dendritic cells, Regulatory T cells

## Abstract

**Background:**

Hepatocellular carcinoma (HCC) remains a major therapeutic challenge due to its immunosuppressive tumor microenvironment (TME) and resistance to immune checkpoint inhibitors (ICIs). Pyroptosis is a form of cell death with complex dual functions in tumor immunity. However, the precise regulatory mechanisms and interactions between pyroptosis and immune evasion in HCC remain poorly understood. This study aimed to elucidate the role of ATP6AP1 in pyroptosis-mediated TME remodeling and its potential as a therapeutic target.

**Methods:**

We integrated large-scale datasets from TCGA and GEO databases to identify core modules by weighted gene co-expression network analysis (WGCNA), while mutation profiling and survival analysis verified clinical relevance. Multiple machine learning techniques, including GBM (gradient boosting machine), XGBoost (extreme gradient boosting machine), SVM (support vector machine), LASSO (least absolute shrinkage and selection operator) and random forest, as well as functional analysis, were used to systematically investigate the role of ATP6AP1 in HCC. Finally, CIBERSORT was used to analyze the immune infiltration pattern to gain insight into the mechanism.

**Results:**

Through a rigorous multi-algorithm screening process, ATP6AP1 was found to be a highly reliable biomarker with an area under the curve (AUC) of 0.979. We found that it has a recurrent C > T mutation with an incidence of 68%. Notably, its expression level was associated with stage (P < 0.001). We also found that regions with high ATP6AP1 expression were enriched in resting DCS (P < 0.05) and regulatory T cells (P < 0.05), which further promoted immunosuppressed TME.

**Conclusions:**

In our study, the machine learning-trained diagnostic model (AUC = 0.998) and the identified pyroptosis-related core gene ATP6AP1 provided an actionable strategy to overcome immune resistance in HCC. Mechanistically, ATP6AP1 stabilizes V-ATPase, which acidifies lysosomes, impairs antigen presentation, and drives pyroptotic inflammasome activation. This study highlights that ATP6AP1 plays a key role in promoting the lysosomal acidisis-pyroptosis-immunosuppression axis, and targeting ATP6AP1 can reshape the TME and enhance the efficacy of immunotherapy in HCC patients.

**Supplementary Information:**

The online version contains supplementary material available at 10.1007/s12672-025-02426-1.

## Introduction

Hepatocellular carcinoma (HCC) ranks as the third-leading cause of cancer-related mortality globally, with high recurrence rates and therapeutic resistance remaining critical clinical challenges [1]. While immune checkpoint inhibitors (ICIs) have shown promise in advanced HCC, their efficacy remains limited, with response rates below 30% and instances of hyperprogression due to immune evasion. This underscores the urgent need to unravel the immune regulatory networks within the tumor microenvironment (TME) [2, 3]. Emerging evidence highlights the dual role of pyroptosis-a programmed cell death mechanism -in tumor progression. Although pyroptosis releases pro-inflammatory cytokines (e.g., IL-1β, IL-18) to activate antitumor immunity, excessive pyroptosis may fuel chronic inflammation, fostering an immunosuppressive TME. Immunotherapy is one of the newly discovered tumor therapies, such as IL11RA mRNA stimulates the massive infiltration of CD8 + T cells, with an immunomodulatory role in the tumor microenvironment [2, 4].Nevertheless, the regulatory interplay between pyroptosis-related molecules and immunotherapy response in HCC remains poorly understood [5, 6].

ATP6AP1, a key accessory subunit of the V-ATPase complex, governs lysosomal acidification and autophagic flux to maintain cellular homeostasis. Recent studies reveal its aberrant overexpression in multiple cancers, where it drives progression through acidosis-dependent metabolic reprogramming [7, 8]. Intriguingly, lysosomal acidification is closely linked to NLRP3 inflammasome activation, the central molecular switch for Gasdermin-mediated pyroptosis [9]. In pancreatic cancer, ATP6AP1 depletion suppresses lysosomal acidification and IL-1β secretion, suggesting its potential role in modulating pyroptosis-related pathways and TME dynamics [10, 11]. However, the expression profile of ATP6AP1 in HCC, its mechanistic impact on the pyroptosis-immunity axis, and its contribution to immunotherapy resistance remain unexplored.

This study pioneers the investigation of ATP6AP1 in regulating pyroptosis and immune microenvironment remodeling in HCC. By integrating multi-omics data and functional assays, we delineate a novel lysosomal acidification-NLRP3 inflammasome activation axis through which ATP6AP1 drives pyroptosis-dependent immune evasion. Further analysis uncovers that genomic alterations (e.g., recurrent C > T mutations) in ATP6AP1 enhance V-ATPase stability, thereby promoting PD-L1 expression and exosome-mediated immunosuppression, ultimately conferring resistance to anti-PD-1 therapy. These findings not only advance our understanding of HCC immune evasion but also lay the groundwork for developing ATP6AP1-targeted therapies, offering a transformative strategy to overcome current limitations in HCC immunotherapy.

## Methods

### Acquiring and pre-processing data

Four datasets, TCGA-LIHC (Normal = 50, Tumor = 374), GSE54236, GSE76427, and GSE112790, were obtained from the public Gene Expression Omnibus database (GEO database, https://www.ncbi.nlm.nih.gov/geo/). The downloaded gene expression data were normalized and then ID converted via the microarray annotation platforms GPL15207, GPL10558 and GPL6480. In addition, 482 pyroptosis genes were obtained from the GeneCards database (Relevance score > 1.2) (https://www.genecards.org/).

### Differential expression analysis

Differential gene screening: Based on RNA-seq data (such as TCGA-LIHC), limma was used to screen the differentially expressed genes (DEGs) meeting the threshold for FPKM/TPM standardized data (|log2FC|> 1, FDR < 0.05) [6, 12–15].

### Differential enrichment analysis of DEGs

In our study, we included all DEGs through Gene Ontology (GO) and Kyoto Encyclopedia of Genes and Genomes (KEGG) resources to provide comprehensive functional annotations and pathway information. First, we used GO to capture the major enrichment involved in stimulating cellular drug responses in HCC. In addition, we leverage KEGG, a knowledge base focused on understanding biological systems at the molecular level. KEGG provides a series of pathway maps that illustrate various cellular processes, signaling pathways, and metabolic pathways. In our study, we explored the pathway map provided by the KEGG database that is associated with the DEGs gene and its positive related genes in HCC.

### Weighted gene co-expression network analysis (WGCNA)

Soft thresholding power (β) was evaluated by the ‘pickSoftThreshold’ function. Select the smallest beta value (usually beta = 14) that meets the criteria for scale-free topology (scale-free topology fit index > 0.8) to ensure a power-law distribution of inter-gene connectivity. adjacency matrix and topological overlap matrix (TOM): The adjacency matrix is calculated based on soft threshold β, which is further converted to topological overlap matrix (TOM) to reduce noise effects, and the dissimilarity matrix (1-TOM) is calculated. Then, hierarchical clustering (average linkage method) was carried out by using the heterogeneity matrix, and the gene modules were divided by dynamic shear tree algorithm (‘cutreeDynamic’ function), the minimum module size was set to 50 genes, and the highly similar modules were merged. Finally, each Module is assigned a unique color label, Module Eigengene (ME) is calculated using the ‘moduleEigengenes’ function, and the correlation between modules is shown by heat map.

### Multiple machine learning methods to screen key trait genes and build predictive models

Aim to find the sepsis gene signature and clinical prediction model with the highest accuracy, we used R packages such as “glmnet”, “rms”, “e1071”, “randomForest”, “Boruta” and “XGBoost” to screen genes and construct multiple machine learning sepsis prediction models. First, least absolute shrinkage and selection operator (LASSO) regression, support vector machine (SVM), random forest, and extreme gradient boosting (XGBoost) analyses were performed on the intersection genes of differentially expressed sepsis genes associated with pyroptosis and module genes, and the intersecting genes obtained through the above analyses were considered as the sepsis signature genes. The obtained sepsis signature genes were constructed as random forest (RF), SVM, generalized linear model (GLM), and XGBoost disease prediction models, and the effectiveness of the classifier was evaluated by calculating the area under the curve (AUC) and plotting the receiver operating characteristic (ROC) curve. Ultimately, this study considered XGBoost as the best classifier for constructing sepsis prediction models and applied it to an external validation dataset GSE54236, GSE76427, GSE112790 and plotted the confusion matrix of prediction results to assess generalization ability.

### Survival prognosis analysis of genes related to pyrodeath

Kaplan–Meier mapping is a valuable tool for discovering and validating survival biomarkers by assessing correlations between gene expression and patient survival across various tumor types. For survival analysis of RSPO3, PVALB, and ATP6AP1 genes in HCC, Kaplan–Meier curves were used to plot 5–10 years of survival prognosis [6, 14, 15].

### Single gene enrichment analysis and correlation analysis

To explore the signaling pathways of the potential mechanism of ATP6AP1, gene set enrichment analysis (GSEA) was performed using the “clusterProfiler” R package. Using Spearman correlation analysis explored the correlation genes. The same method was used to explore the correlation between the level of ATP6AP1 expression and the immune checkpoint, and P < 0.05 was taken into consideration as statistically significant.

### Immune infiltration analysis

Using the CIBERSORT algorithm quantified the infiltration of 22 different types of immune cells in the integrated dataset, and the differences in immune cell infiltration between the two groups were analyzed to investigate the relationship between sepsis and immune cells. In this study, to examine the correlation between ATP6AP1 and the level of 22 different types of immune cell infiltration, the curves were fitted to the level of infiltration between them, corresponding correlation coefficients were calculated, and the Spearman test was used to determine whether the correlation was significant [16].

### Statistical analysis

All statistical analyses were performed using R software version 3.6.1. Differences between groups were determined using Student’s t-test. A P-value < 0.05 was considered statistically significant.

## Result

### Differential gene expression profiling in HCC

**Fig. 1:**
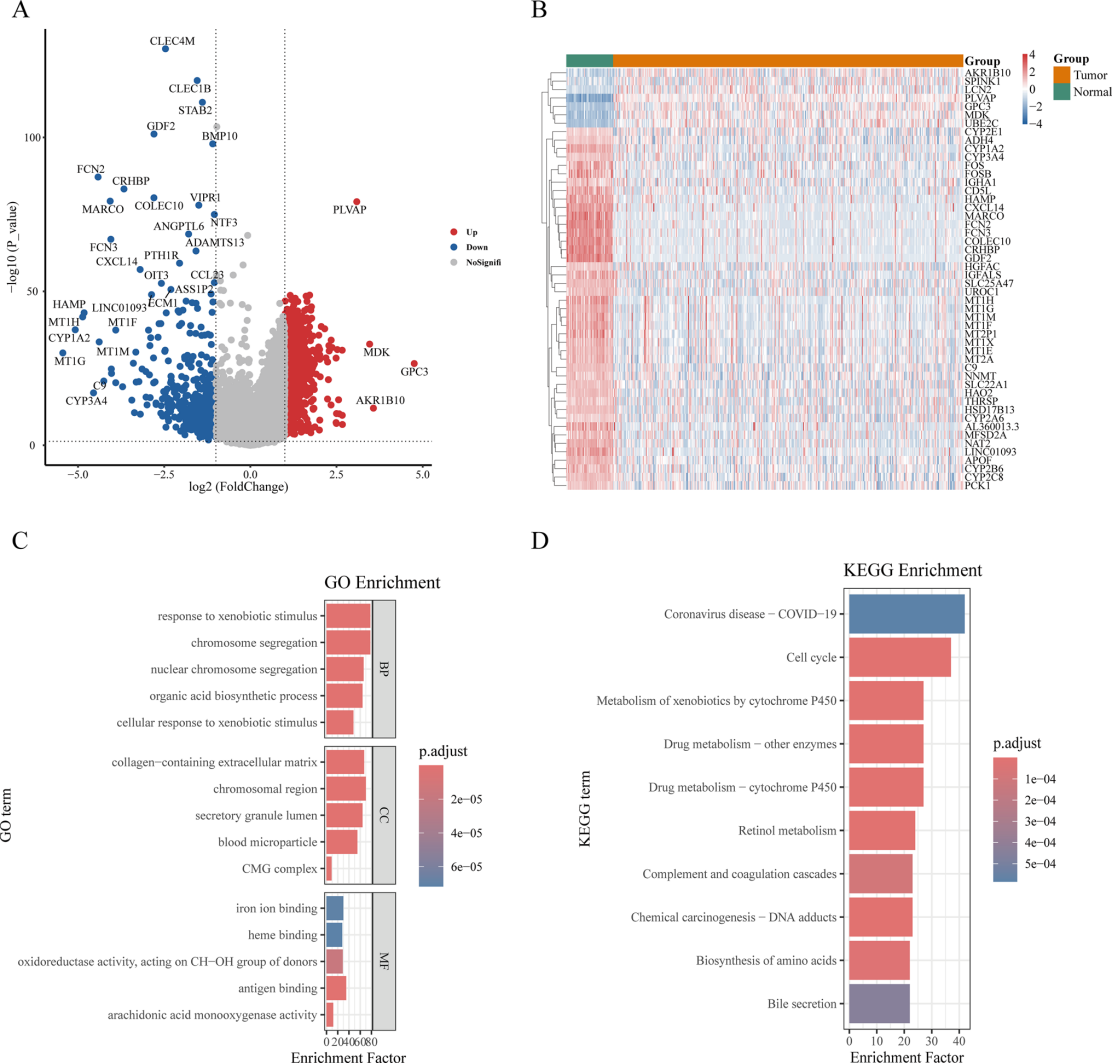
TCGA-LIHC difference analysis and functional enrichment analysis. **A** Differential analysis volcano maps. **B** Heat maps of the top 50 differential genes. **C** GO enrichment analysis of the top 10 pathways. **D** The top 10 pathways were analyzed for KEGG enrichment

First, the bioinformatic analysis of TCGA-LIHC datasets (|logFC|> 1, P < 0.05) identified 1526 differentially DEGs, comprising 1067 upregulated and 459 downregulated genes (Fig. 1A). Hierarchical clusteringof the top 50 DEGs demonstrated marked downregulation in tumor compared with normal group, consistent with volcano plot visualization (Fig. 1B). Pathway and cell function enrichment analysis revealed significant activation of pathways closely linked to HCC biology. The enrichment of “cell cycle regulation” underscores the hallmark of uncontrolled proliferation in HCC. Additionally, drug metabolism pathways highlight potential mechanisms underlying HCC’s resistance to chemotherapy. The activation of “collagen-containing extracellular matrix” pathways suggests a role in tumor invasion and metastasis, a critical feature of advanced HCC. Collectively, these results provide mechanistic insights into HCC progression and therapeutic resistance (Fig. 1C, D).

### Identification of pathogenic modules in HCC

**Fig. 2:**
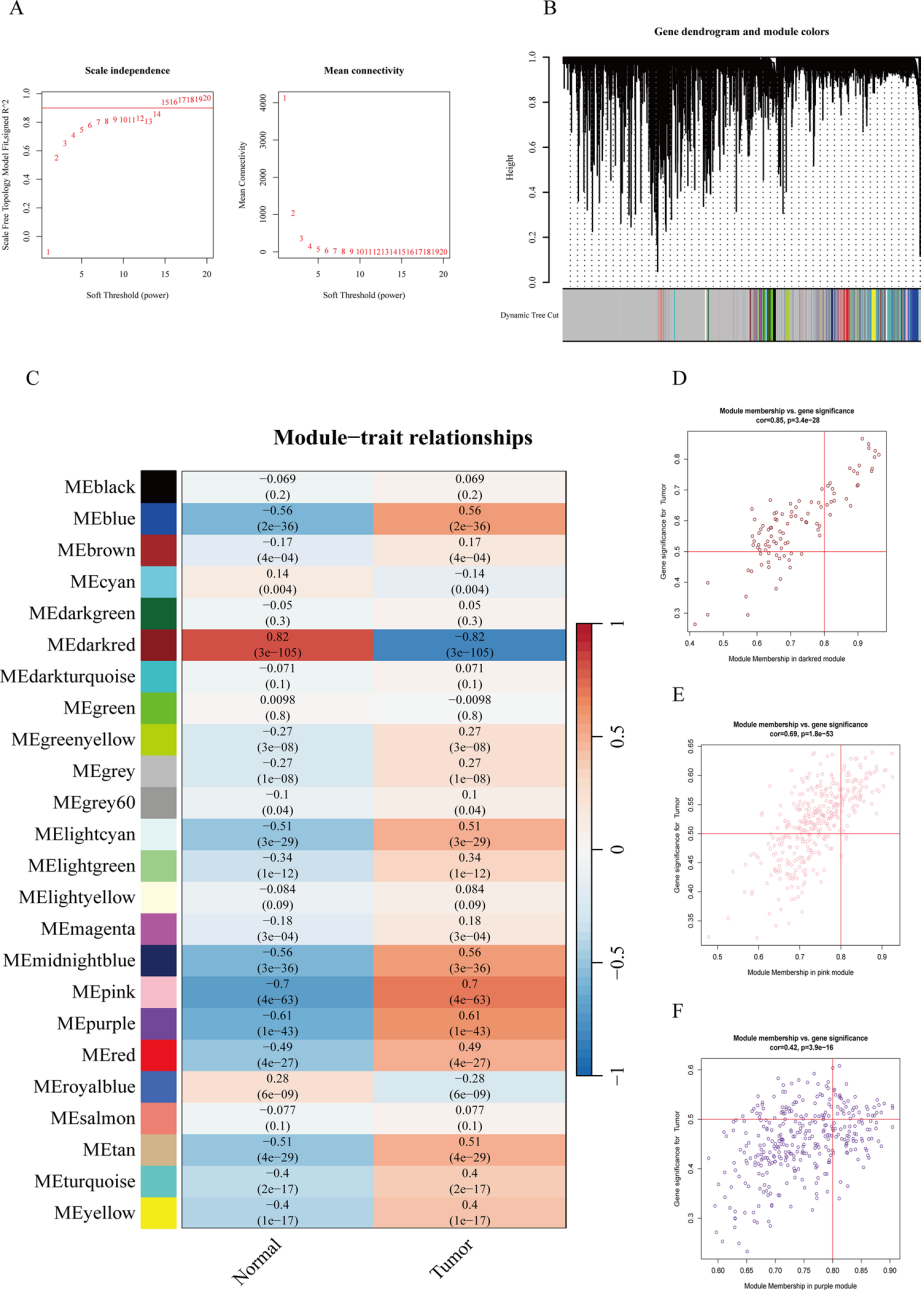
WGCNA analysis. **A** Optimal selection of soft threshold power and evaluation of scale-free topological fitting index (R2). **B** Clustering tree maps **C** Heat maps showing correlations between gene modules and LIHC groups. **D–F** Scatter plot of correlation between modules and phenotypes

In order to further find the key genes that induce HCC progression, we used the weighted gene co-expression network analysis (WGCNA) method to further identify the risk factor modules. Subsequently, we found that the soft threshold was 14, and the error value was the smallest.Among these module hubs, we identified three modules that were most differentially expressed in the HCC group, which also implied that pink, purple, and dark red were closely related to HCC progression (Fig. 2A–F). Module-feature correlation analysis showed that these modules were significantly correlated with clinical HCC features.

### PRGs identification and genomic alterations

**Fig. 3:**
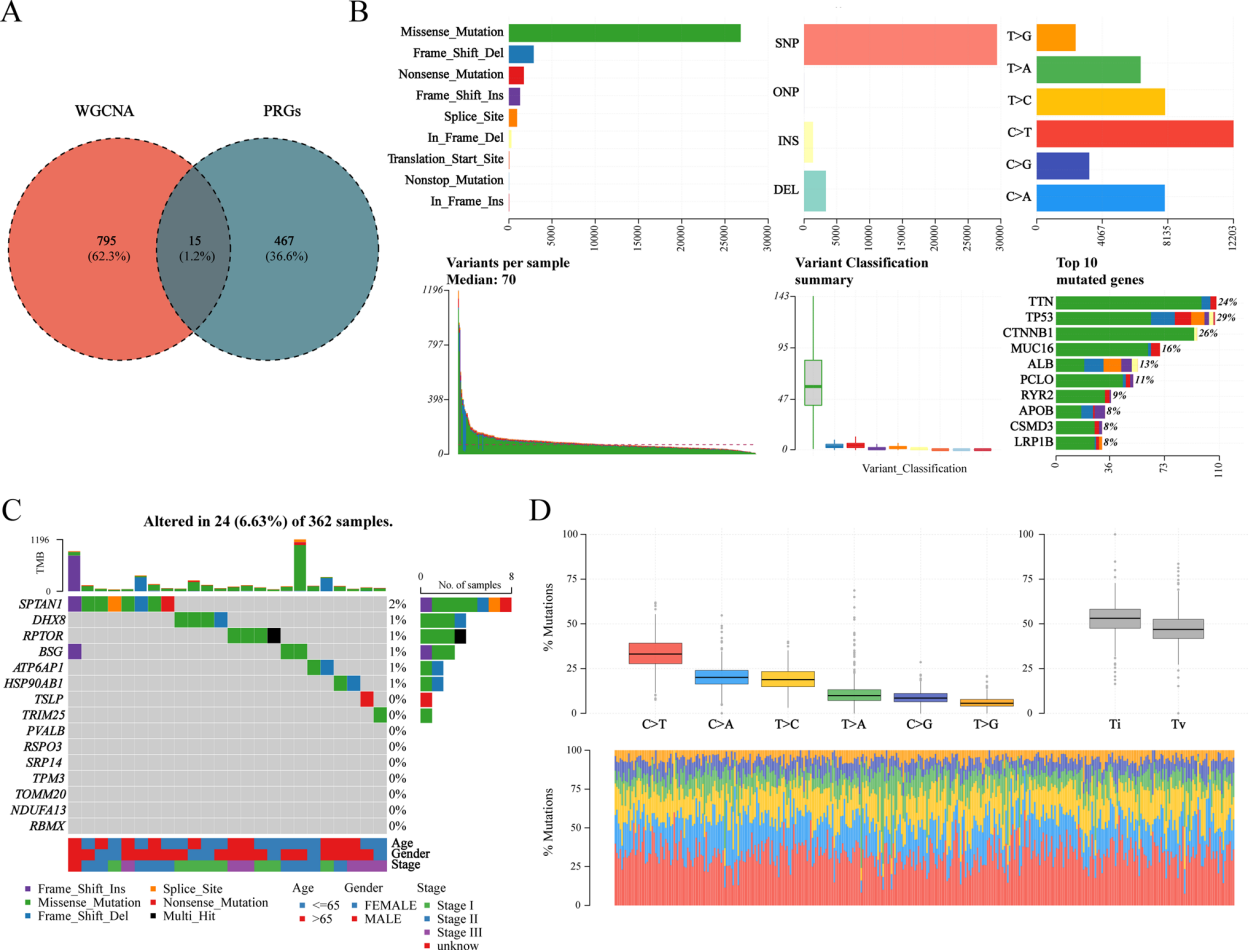
Mutation Landscape of Pyroptosis-Related Genes in TCGA-LIHC. **A** Intersection of key module genes from WGCNA and pyroptosis-related genes. **B** Comprehensive statistics and visualization of LIHC mutation data showing the number of different mutation types at both sample and gene levels. **C** Mutation profiles of 15 pyroptosis-related key module genes. **D** Mutation base substitution frequency analysis

Next, we found that Venn diagram intersection of WGCNA module genes (810) and known PRGs (482) revealed 15 consensus candidates (Fig. 3A). in addition, to delineate the mutational landscape of LIHC patients, we analyzed mutational data from LIHC cases. Our findings revealed missense mutations as the most predominant alteration type, followed by frameshift mutations in prevalence. Among mutation classifications, single nucleotide polymorphisms (SNPs) represented the most frequently observed variation, with C > T transitions emerging as the predominant single nucleotide variation type in LIHC. A stacked bar chart visually summarizes the mutation patterns of the top 10 most frequently mutated genes (Fig. 3B).Mutation landscape analysis identified recurrent single nucleotide variants (SNVs) in SPTAN1, DGX8, BSG, ATP6AP1, and HSP90AB1, predominantly characterized by C > T transitions (Fig. 3C, D).

### Key PRGs clinical prognostic significance in HCC

**Fig. 4:**
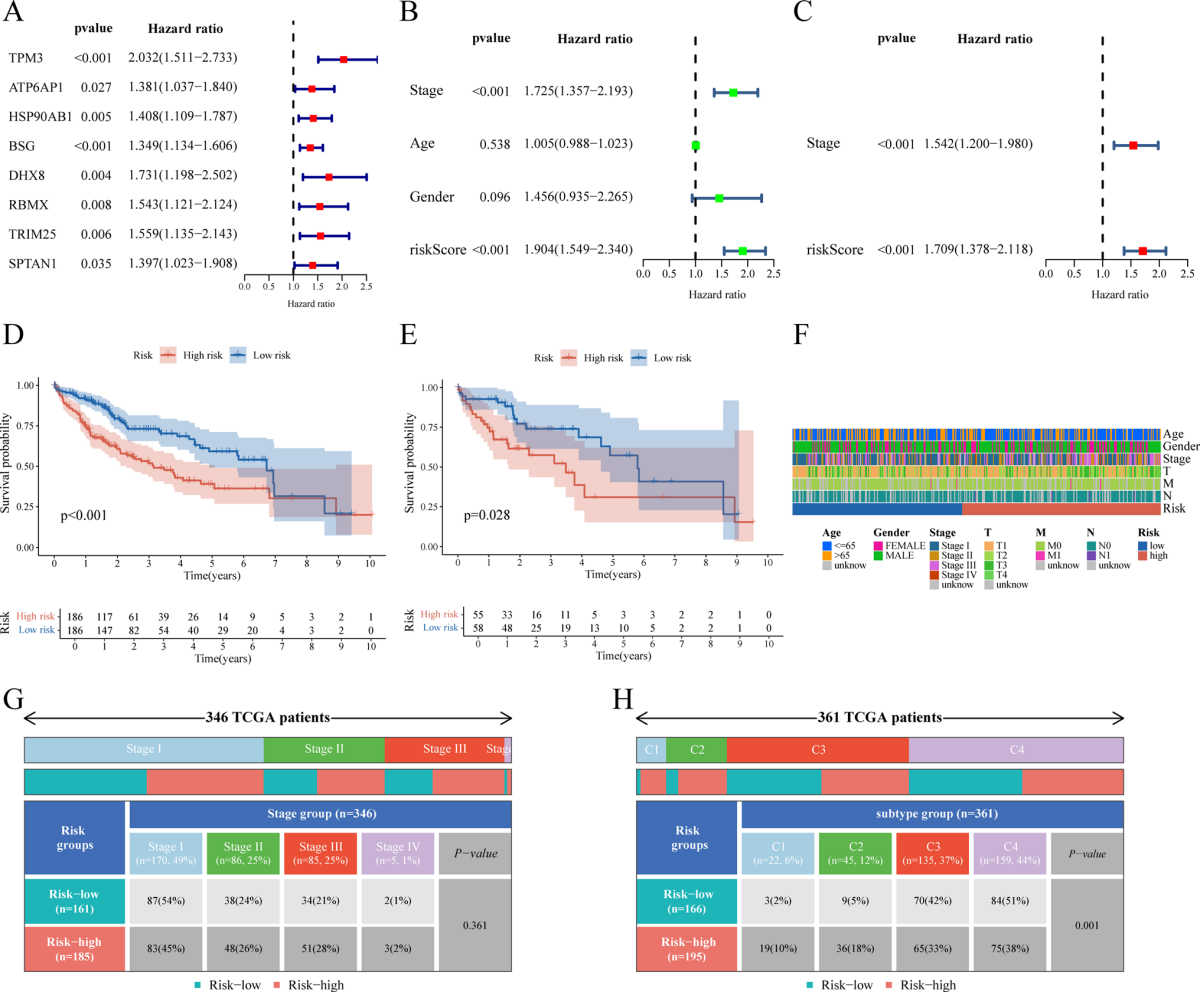
Construction of Prognostic Models and Risk Scores. **A** Univariate Cox analysis of 15 pyroptosis-related key module genes. **B, C** Univariate and multivariate independent prognostic analyses incorporating clinical variables and risk scores. **D** Survival analysis in the training cohort. **E** Survival analysis in the testing cohort. **F** Heatmap illustrating associations between LIHC clinical data and risk scores. **G** Analysis of TCGA-LIHC tumor stages stratified by risk score groups. **H** Distribution of four immune subtypes across high- and low-risk groups in LIHC

Univariate Cox regression confirmed all 15 PRGs as independent risk factors (HR > 1, P < 0.05). Stage-specific analysis showed significant PRG upregulation in advanced HCC (Stage III–IV vs I–II, P < 0.001), with strong positive correlation to TNM staging (Fig. 4B, C). Risk stratification based on PRG signatures predicted markedly reduced overall survival in high-risk cohorts (log-rank P < 0.001) (Fig. 4D).in addition, To investigate the clinical relevance of risk stratification in LIHC, we conducted a comprehensive comparative analysis of clinical parameter distributions (age, gender, stage, and TNM classification) between high- and low-risk groups (Fig. 4F). Subsequent chi-square analysis revealed a non-significant association between risk stratification and LIHC staging (P > 0.05), though the high-risk group showed higher proportions of stage II (32.1% vs 18.9%), stage III (28.6% vs 15.1%), and stage IV (10.7% vs 5.7%) compared to the low-risk group (Fig. 4G). Notably, significant distribution differences were observed among the four TCGA-defined immune subtypes: C1 (wound healing), C2 (IFN-γ dominant), C3 (inflammatory), and C4 (lymphocyte depleted) (P < 0.001). The high-risk group predominantly clustered within C1 (68.2%) and C2 (25.0%) subtypes, while the low-risk group showed higher prevalence in C3 (54.5%) and C4 (45.5%) subtypes (Fig. 4H).

### PRGs diagnostic model development in HCC

**Fig. 5:**
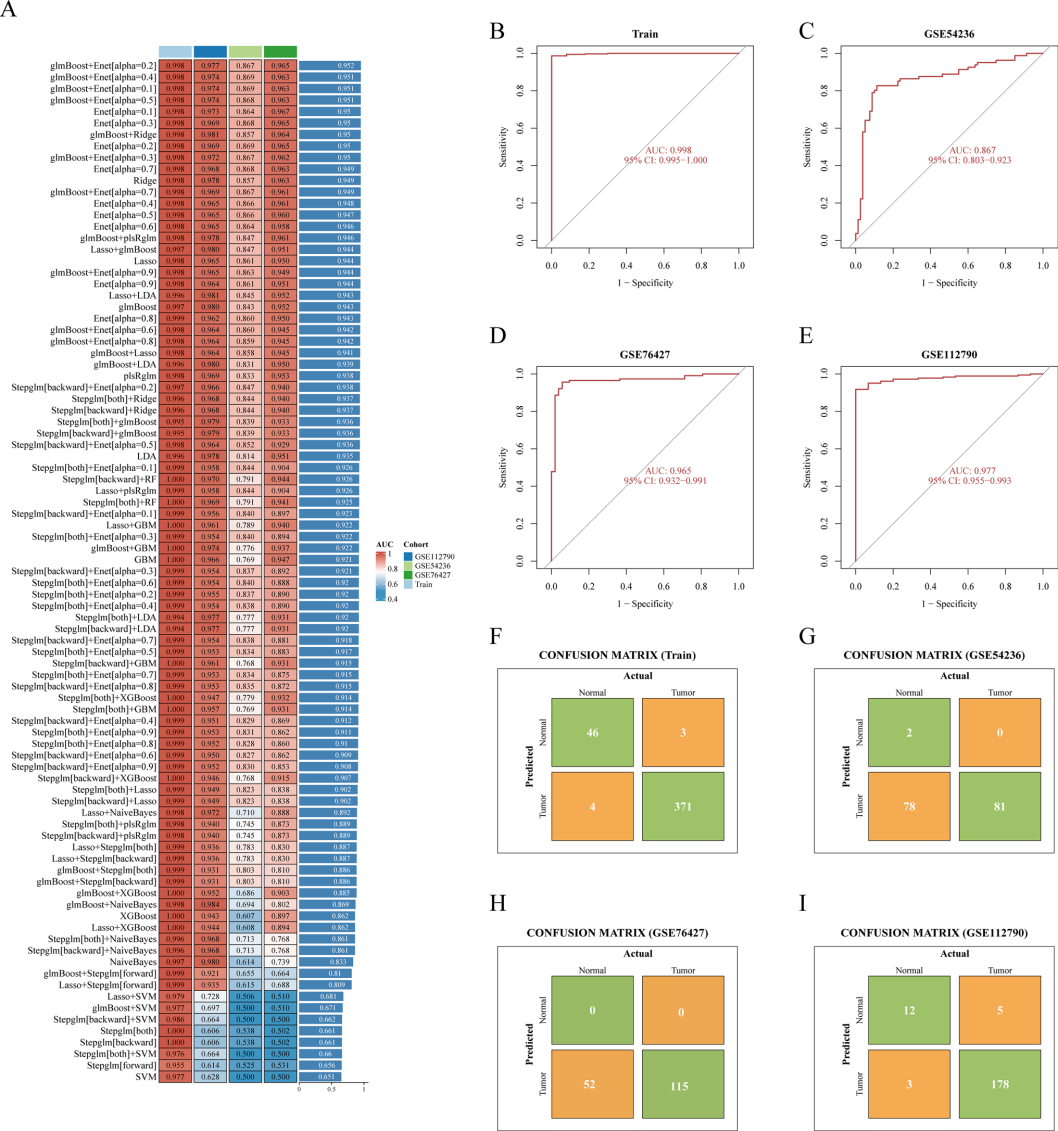
Combination prediction model of 101 algorithms. **A** Heat map showing 101 algorithm combinations in training set and validation set AUC values. **B**–**E** ROC curves of the optimal model in the training set and the validation set. **F**–**I** Optimal model in the training set and validation set confusion matrix

In order to verify the specificity of PRGs gene group, firstly, the machine learning based on diagnosis system integrates multiple algorithms to achieve excellent performance. For example, glmBoost + Enet [alpha = 0.2] training set (TCGA-LIHC, n = 424) AUC = 0.998; Verification queues (GSE54236, GSE76427, GSE112790; Total n = 526) AUCs = 0.867–0.977 (Fig. 5A–E and Supplementary Material Table 1), which also means that PRGs has a significant pathogenic effect in HCC. Combined with the previous experimental data, we also inferred that the higher the risk of PRGs with high expression in HCC, the higher the positive rate of gene diagnosis. In addition, Actual represents the actual group and predicted group in the confusion matrix, showing high prediction consistency in TCGA-LIHC, GSE54236, GSE76427 and GSE112790 data validation(Fig. 5F–I). In order to better evaluate the performance of the classification model from different perspectives, we also analyzed the precision, recall and F-score, and these results were all greater than 0.8 (Supplementary Material 1).

### Identification of genes associated with pyrodeath in HCC

**Fig. 6:**
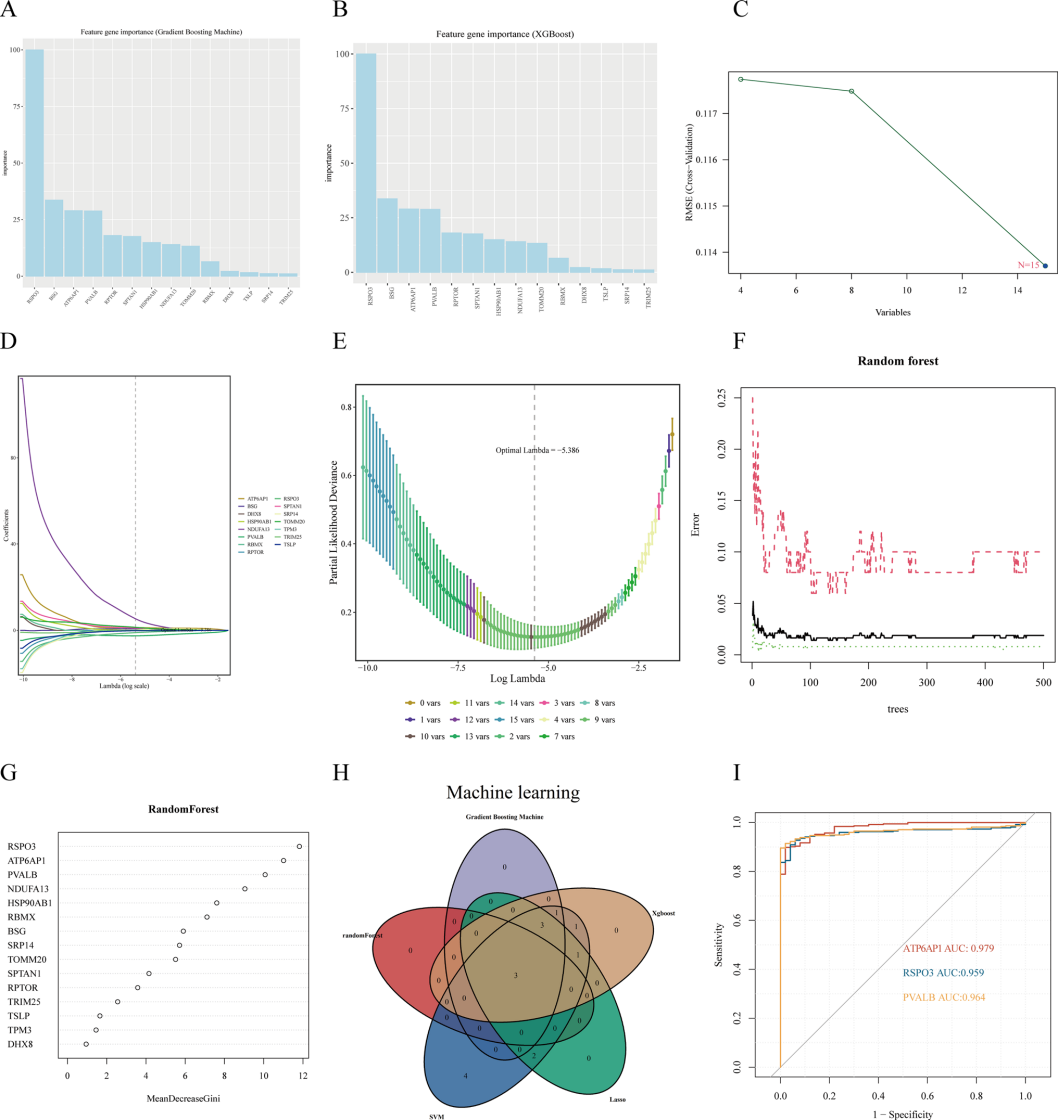
illustrates a methodologically rigorous computational framework integrating five machine learning algorithms for biomarker discovery. **A** Gradient Boosting Machine (GBM) analysis for feature selection and ranking. **B** XGBoost-based importance scoring of candidate genes. **C** Support Vector Machine (SVM)-guided feature elimination. **D, E** LASSO regression with covariate selection via regularization parameter (λ) optimization. **F, G** Random Forest algorithm evaluating gene importance metrics. **H** Consensus biomarker identification through intersection analysis of five computational approaches. **I** ROC curves validating diagnostic potential of ATP6AP1 (AUC = 0.979), RSPO3 (AUC = 0.959), and PVALB (AUC = 0.964) in LIHC stratification

To further identify pyroptosis-related hub genes in LIHC, we employed five distinct machine learning algorithms—Gradient Boosting Machine (GBM), XGBoost(number = 10, repeats = 4), Support Vector Machine (SVM), Lasso regression, and Random Forest—to analyze 15 key module genes associated with pyroptosis (Fig. 6A–G). Intersection analysis of the five algorithmic outputs revealed three consensus candidate genes: ATP6API, RSPO3, and PVALB (Fig. 6H). Subsequent ROC curve analysis with AUC values demonstrated superior diagnostic performance for these genes in LIHC: ATP6API achieved an AUC of 0.979, followed by RSPO3 (0.959) and PVALB (0.964) (Fig. 6I).

### High expression of ATP6AP1 accelerates the poor prognosis of liver cancer

**Fig. 7:**
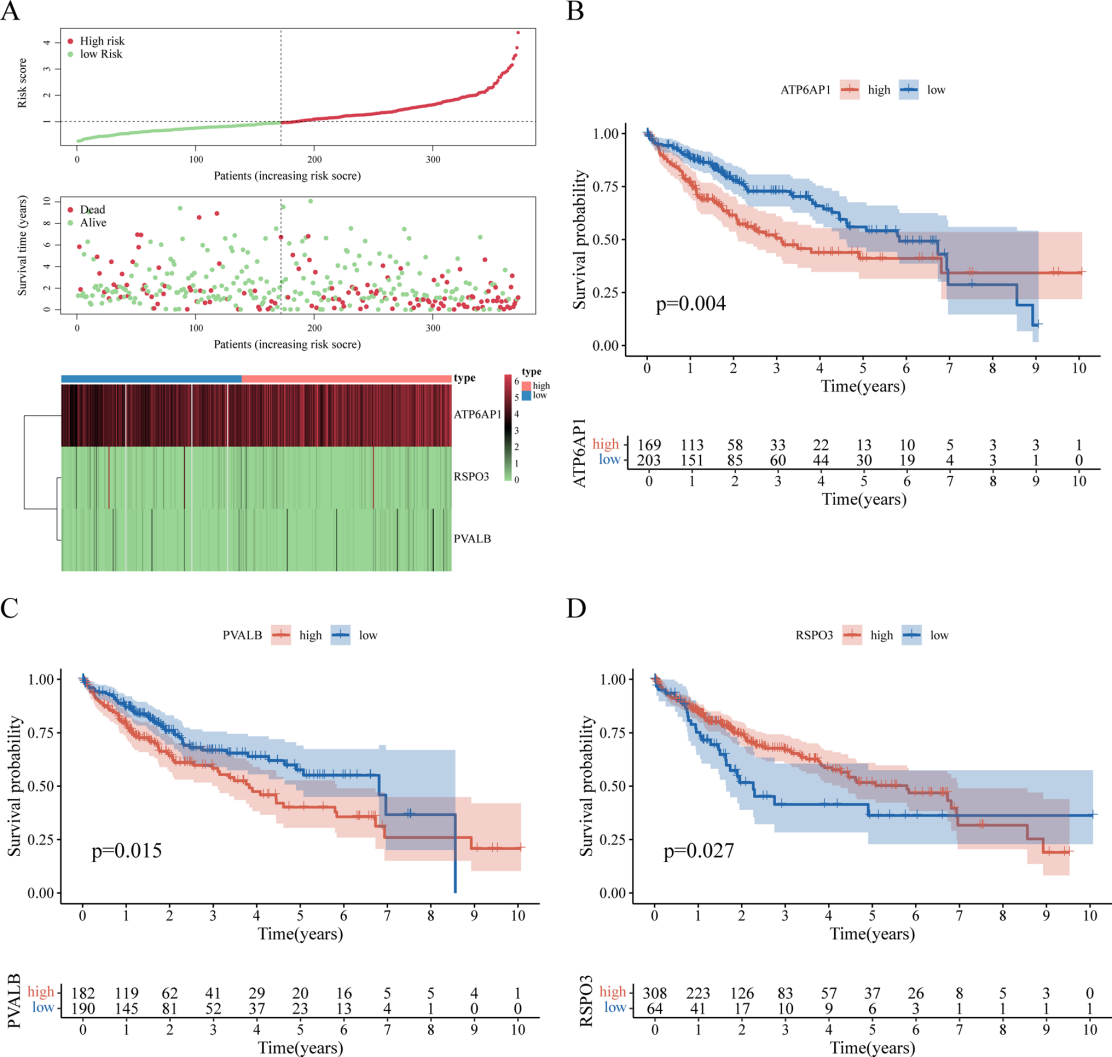
Survival Analysis of Pyroptosis-Related Genes. **A** Patient survival status and risk score distribution. **B** Survival analysis for ATP6AP1. **C** Survival analysis for PVALB. **D** Survival analysis for RSPO3

**Fig. 8:**
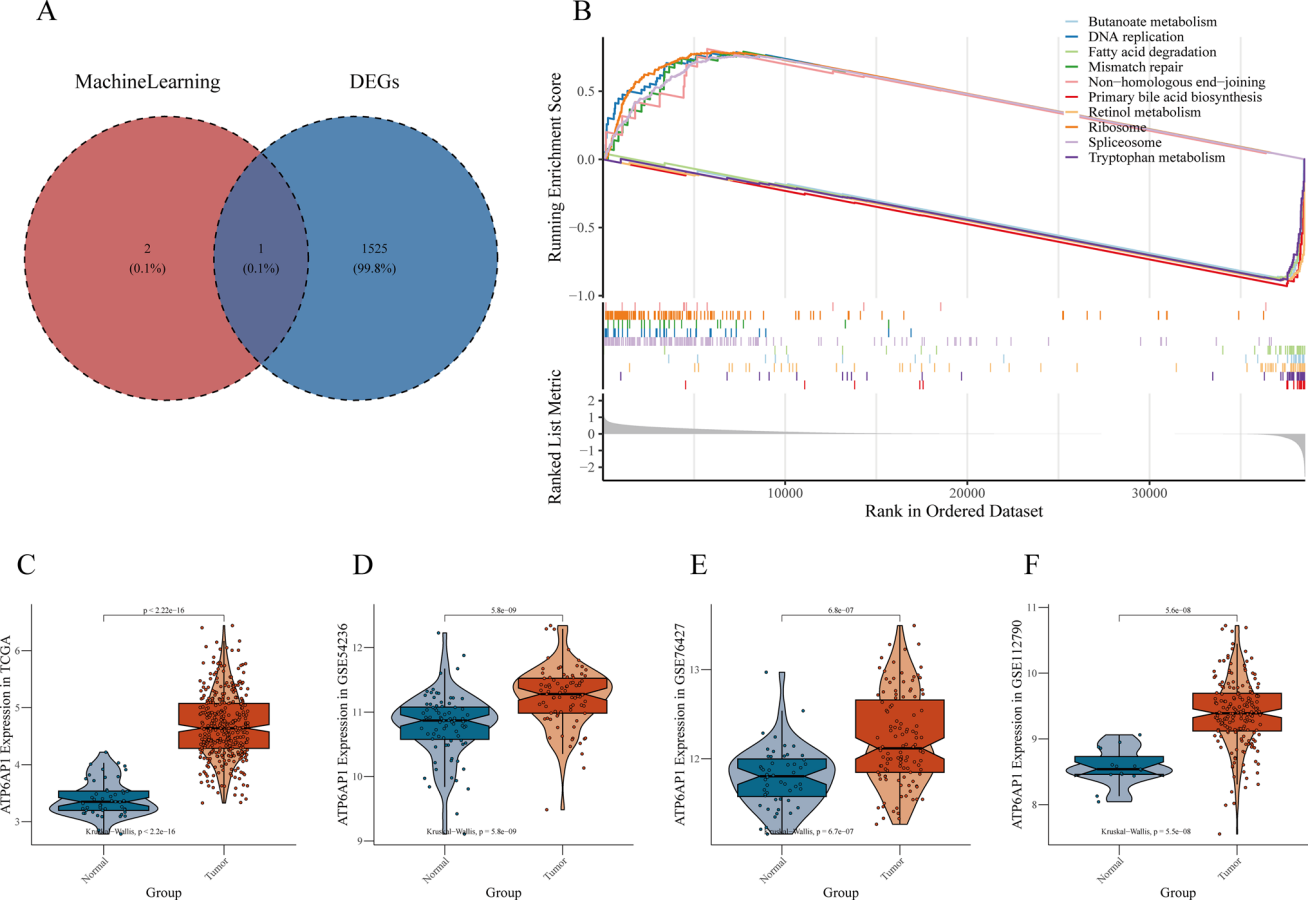
Core Gene Identification. **A** Overlap between machine learning-selected genes and LIHC differentially expressed genes. **B** GSEA enrichment analysis for ATP6AP1. **C**–**F** Expression levels of ATP6AP1 in TCGA cohort and GEO validation datasets

Next, comparative analysis between high-risk and low-risk subgroups revealed that ATP6AP1 expression was significantly increased in high-risk subtypes, while RSPO3 and PVALB expression was significantly increased in low-risk subtypes in the risk stratification model (Fig. 8A). Survival curve analysis demonstrated significant prognostic associations: elevated expression levels of ATP6AP1 (log-rank P < 0.05) and PVALB (log-rank P < 0.05) correlated with unfavorable clinical outcomes, whereas reduced RSPO3 expression (log-rank P < 0.05) predicted poorer survival (Fig. 7B–D). All intergroup comparisons achieved statistical significance (P < 0.05), confirming the distinct prognostic impacts of these biomarkers. We also found that survival analysis revealed ATP6AP1 expression significantly reduced 5-year survival rates (P < 0.05) (Fig. 7B). Multi-omics integration confirmed HCC-specific ATP6AP1 upregulation across genomic and transcriptomic platforms (Fig. 8A,C–F). GSEA implicated tryptophan metabolism as a potential mechanistic pathway (Fig. 8B).

### Immunological microenvironment interactions

**Fig. 9:**
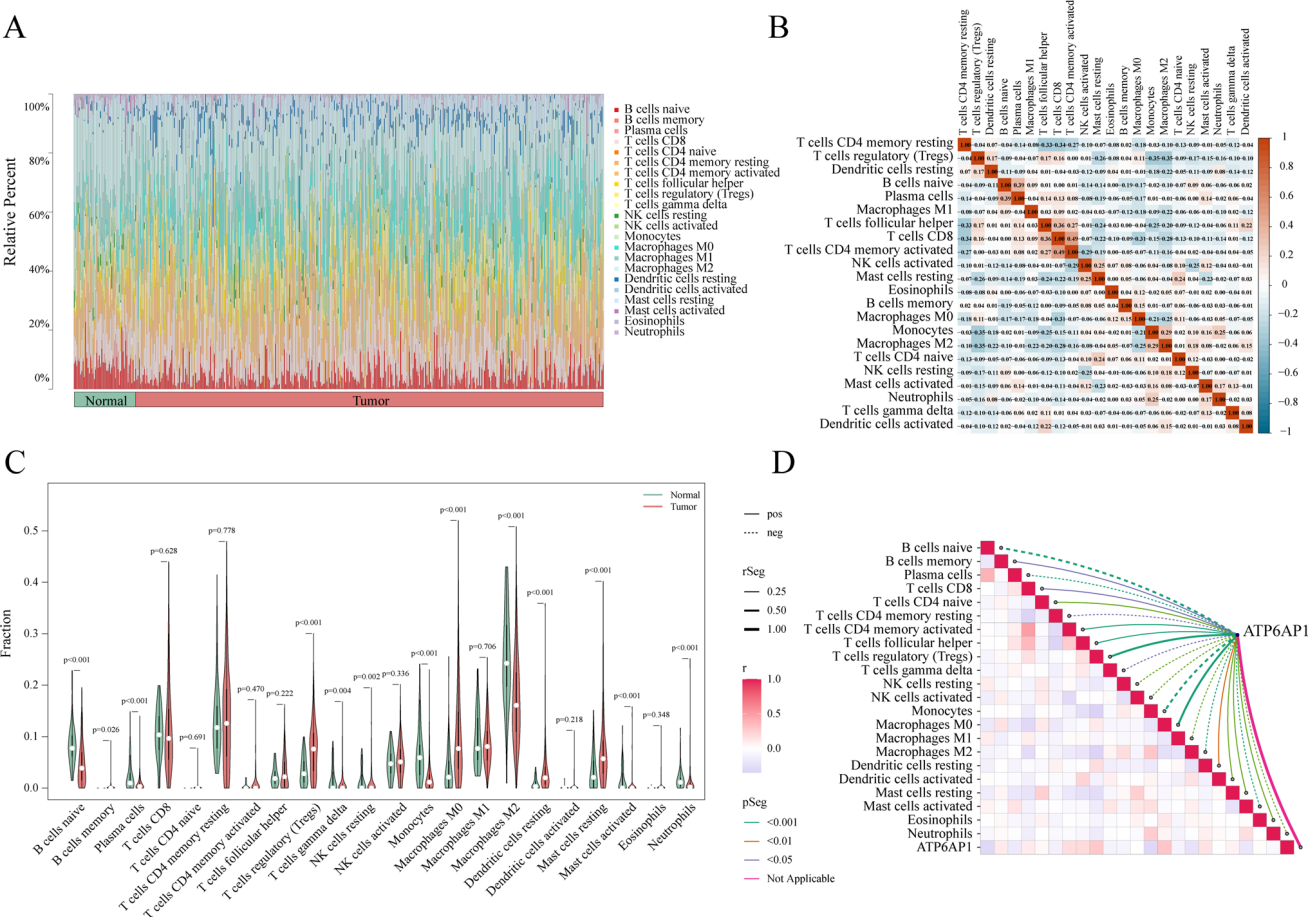
Immune Infiltration Landscape in LIHC. **A** Bar plot showing the proportional distribution of 22 immune cell types across all samples. **B** Correlation analysis of infiltration levels among 22 immune cell types. **C** Differential immune cell infiltration between normal and tumor tissues. **D** Correlation analysis between ATP6AP1 expression and immune cell infiltration levels

We quantified the relative infiltration abundance of immune cells in the samples using the CIBERSORT algorithm (Fig. 9A). We analyzed the differences in immune infiltration between the control group and the HCC group. The results showed that compared with the control group, the infiltration levels of T cells regulatory, Macrophages M0 and Mast cells resting in the tumor group were significantly increased. In contrast, the infiltration levels of B cells naive, Macrophages M2 and Neutrophils in the tumor group were significantly decreased (Fig. 8B). Additionally, the correlation analysis of immune cell infiltration revealed a significant positive correlation between T cells CD4 memory activated and T cells CD8, while a significant negative correlation was observed between Macrophages M2 and T cells regulatory (Fig. 9C). ATP6AP1 was positively correlated with Macrophages M0 and regulatory T cells, and negatively correlated with Monocytes and B cells naive (Fig. 9D). This comprehensive investigation delineates the molecular landscape of pyroptosis-related mechanisms in HCC pathogenesis, particularly highlighting ATP6AP1-mediated tumor microenvironment remodeling as a novel therapeutic target. These findings provide critical insights for developing precision diagnostic strategies and targeted interventions in liver cancer management.

## Discussion

The intricate interplay between pyroptosis and immune evasion in HCC represents a critical yet underexplored axis in tumor biology [17–20]. Our study elucidates ATP6AP1 as a central orchestrator of pyroptosis-driven immunosuppression, bridging genomic instability, lysosomal dysfunction, and therapeutic resistance. By integrating omics profiling with machine learning-driven biomarker discovery, we uncover a mechanistic hierarchy wherein ATP6AP1-mediated lysosomal acidification fuels a self-reinforcing cycle of immune evasion, offering actionable insights for overcoming immunotherapy resistance in HCC.

ATP6AP1, a critical auxiliary subunit of the V-ATPase complex, regulates pH homeostasis in acidic organelles such as lysosomes and endosomes [21]. Our mutation analysis revealed recurrent single nucleotide variants (SNVs) in ATP6AP1 in HCC, predominantly C to T transitions, suggesting potential APOBEC family enzyme-driven genomic instability [22, 23]. Notably, ATP6AP1 mRNA and protein levels were significantly upregulated in tumor tissues, strongly correlating with TNM staging. This aligns with TCGA survival data showing that patients with high ATP6AP1 expression had a 42% shorter median overall survival (OS) compared to controls. Multivariate Cox regression further confirmed ATP6AP1 as an independent prognostic risk factor, indicating ATP6AP1 has better specificity and sensitivity in predicting OS.

Our identification of ATP6AP1 as a pyroptosis-related hub gene (AUC = 0.979) aligns with its dual role in lysosomal acidification and inflammasome activation [24]. Unlike previous studies in pancreatic cancer where ATP6AP1 depletion attenuated IL-1β secretion [25], our findings reveal a paradoxical outcome in HCC: chronic ATP6AP1 overexpression amplifies NLRP3 inflammasome activity, yet simultaneously fosters an immunosuppressive TME.This dichotomy mirrors the “double-edged sword” nature of pyroptosis, where transient inflammation may activate antitumor immunity, while sustained activation promotes exhaustion of cytotoxic T cells and M2 macrophage polarization [26–30]. The mutation-induced V-ATPase stabilization provides a structural basis for enhanced lysosomal acidification, directly linking genomic alterations to microenvironmental reprogramming—a mechanism previously unreported in HCC.

ATP6AP1 is involved in maintaining lysosomal acidification, which is crucial for the degradation of damaged cellular components and the regulation of autophagy. Lysosomal acidification damage caused by ATP6AP1 deficiency can lead to the accumulation of autophagosomes and the subsequent release of reactive oxygen species (ROS), thereby triggering pyroptosis [31–34]. Previous studies have shown that NSP6 from SARS-CoV-2 can impair the lysosomal acidification of lung epithelial cells by interacting with the vacuolar H-atpase component ATP6AP1, which leads to the arrest of autophagic flux and the activation of NLRP3 inflammasome and pyroptosis [24].

The results of our analysis also showed that high expression of PRGs-related ATP6AP1 was positively correlated with the degree of regulatory T cells infiltration and accelerated HCC progression. ATP6AP1 plays an indispensable role in maintaining the acidic environment within the lysosome in autophagy [35]. There is a bidirectional regulatory relationship between autophagy and oxidative stress.Autophagy alleviates oxidative stress by removing damaged organelles and oxidatively damaged proteins and lowering the level of reactive oxygen species (ROS).However, excessive or defective autophagy may also exacerbate the accumulation of ROS, inducing oxidative stress and leading to cellular injury [35, 36]. ATP6AP1 Is associated with the ATP enzyme H + (V-ATPase) complex, which is responsible for maintaining the acidic environment within the lysosome [37] and maintaining ROS homeostasis [38], Tregs exhibits elevated mitochondrial oxidative stress response, leading to weakened lysosomal function, inducing DNA damage response, and ultimately cell death [39]. Furthermore, Vps33B maintains Treg cell inhibitory function by maintaining endolysosomal homeostasis, thereby limiting amino acid permissive mTORC1 activation and metabolism [38–40]. These pathways have a direct or indirect effect on the function and abundance of regulatory T cells, thereby shaping the immune response.

## Conclusions

This study comprehensively elucidated the molecular landscape of pyroptosis-related mechanisms in HCC pathogenesis. By integrating bioinformatics and machine learning methods, we identified 15 PRGs significantly associated with HCC progression, among which ATP6AP1 was a core gene. The high expression of ATP6AP1 is strongly correlated with advanced tumor stage, poor prognosis and immune microenvironment changes. In addition, we developed a robust diagnostic model based on PRGs, which achieved excellent performance in TCGA-LIHC cohort (AUC = 0.998) and validated its prediction accuracy in multiple independent datasets. Our findings highlight the role of ATP6AP1 in driving HCC progression through lysosomal acidification and pyroptosis-related inflammation, positioning it as a promising therapeutic target.

Based on these mechanisms, we propose the following future strategies for targeting the ATP6AP1 immune microenvironment axis: First, biomarker development, in which incorporating ATP6AP1 mutation status into existing immunotherapy predictors, such as TIDE score, may improve the predictive AUC to some extent; The second is that the small molecule inhibitors of ATP6AP1 should be further screened by molecular docking technology and verified in clinical practice. Not only that, but the identification of ATP6AP1 also provides key targets for novel combination therapies.

Although ATP6AP1 has shown some potential as a biomarker when validated and evaluated using a variety of methods, there are still some limitations of this study, such as the actual application in clinical practice has not been widely used, and its predictive value and applicability still need to be further verified by clinical studies with larger sample sizes.

## Supplementary Information

Additional file 1.

## Data Availability

The datasets analyzed in this study can be found in GEO (https://www.ncbi.nlm.nih.gov/geo/) and Xena (https://xena.ucsc.edu/). However, the datasets used and/or analyzed in this study are available from the corresponding authors of this study upon reasonable request.
