# Supplementary material for: ATP6AP1 drives pyroptosis-mediated immune evasion in hepatocellular carcinoma: a machine learning-guided therapeutic target
Source: Discov Oncol. 2025 Apr 25;16:616. doi: 10.1007/s12672-025-02426-1 (PMC12032091; doi:10.1007/s12672-025-02426-1)
Supplement: Supplementary file 1 — Additional file 1. [file 12672_2025_2426_MOESM1_ESM.docx]

Table1. Specific results for each machine learning training model

|  | **Accuracy** | **Precision** | **Recall** | **F1-score** |
| --- | --- | --- | --- | --- |
| Lasso+Stepglm[both] | 0.844210526 | 0.844141069 | 0.985391766 | 0.909313725 |
| SVM | 0.834736842 | 0.832589286 | 0.990703851 | 0.904790782 |
| glmBoost+SVM | 0.843157895 | 0.840857788 | 0.98937583 | 0.909090909 |
| Ridge | 0.84 | 0.839548023 | 0.986719788 | 0.907203907 |
| Lasso+SVM | 0.846315789 | 0.8421646 | 0.992031873 | 0.91097561 |
| glmBoost+Ridge | 0.842105263 | 0.839909808 | 0.98937583 | 0.908536585 |
| Enet[alpha=0.1] | 0.838947368 | 0.840136054 | 0.984063745 | 0.906422018 |
| glmBoost+Enet[alpha=0.1] | 0.842105263 | 0.840677966 | 0.988047809 | 0.908424908 |
| Enet[alpha=0.2] | 0.844210526 | 0.84180791 | 0.98937583 | 0.90964591 |
| Enet[alpha=0.3] | 0.843157895 | 0.840857788 | 0.98937583 | 0.909090909 |
| glmBoost+Enet[alpha=0.3] | 0.844210526 | 0.842582106 | 0.988047809 | 0.909535452 |
| glmBoost+Enet[alpha=0.2] | 0.843157895 | 0.840857788 | 0.98937583 | 0.909090909 |
| Enet[alpha=0.4] | 0.847368421 | 0.844671202 | 0.98937583 | 0.911314985 |
| glmBoost+Enet[alpha=0.4] | 0.844210526 | 0.842582106 | 0.988047809 | 0.909535452 |
| Lasso+glmBoost | 0.856842105 | 0.853379152 | 0.98937583 | 0.916359164 |
| Enet[alpha=0.5] | 0.847368421 | 0.844671202 | 0.98937583 | 0.911314985 |
| glmBoost | 0.852631579 | 0.850285714 | 0.988047809 | 0.914004914 |
| glmBoost+Enet[alpha=0.5] | 0.845263158 | 0.843537415 | 0.988047809 | 0.910091743 |
| Enet[alpha=0.6] | 0.848421053 | 0.845629966 | 0.98937583 | 0.911872705 |
| glmBoost+Enet[alpha=0.6] | 0.845263158 | 0.842760181 | 0.98937583 | 0.910201588 |
| glmBoost+Enet[alpha=0.7] | 0.845263158 | 0.842760181 | 0.98937583 | 0.910201588 |
| glmBoost+Enet[alpha=0.8] | 0.847368421 | 0.844671202 | 0.98937583 | 0.911314985 |
| Enet[alpha=0.8] | 0.843157895 | 0.840857788 | 0.98937583 | 0.909090909 |
| Enet[alpha=0.9] | 0.844210526 | 0.84180791 | 0.98937583 | 0.90964591 |
| Lasso | 0.846315789 | 0.843714609 | 0.98937583 | 0.910757946 |
| Enet[alpha=0.7] | 0.844210526 | 0.84180791 | 0.98937583 | 0.90964591 |
| glmBoost+Enet[alpha=0.9] | 0.848421053 | 0.845629966 | 0.98937583 | 0.911872705 |
| glmBoost+Lasso | 0.845263158 | 0.842760181 | 0.98937583 | 0.910201588 |
| Lasso+plsRglm | 0.84 | 0.840317101 | 0.985391766 | 0.907090465 |
| glmBoost+plsRglm | 0.834736842 | 0.834080717 | 0.988047809 | 0.904559271 |
| glmBoost+Stepglm[forward] | 0.833684211 | 0.838452787 | 0.97875166 | 0.903186275 |
| Lasso+Stepglm[forward] | 0.831578947 | 0.832772166 | 0.985391766 | 0.902676399 |
| Stepglm[forward] | 0.826315789 | 0.832579186 | 0.977423639 | 0.899205864 |
| plsRglm | 0.835789474 | 0.836527621 | 0.985391766 | 0.904878049 |
| Stepglm[both]+Ridge | 0.835789474 | 0.834266517 | 0.98937583 | 0.905224787 |
| Stepglm[backward]+Ridge | 0.830526316 | 0.829621381 | 0.98937583 | 0.902483343 |
| Stepglm[both]+plsRglm | 0.829473684 | 0.834654587 | 0.97875166 | 0.900977995 |
| Stepglm[backward]+plsRglm | 0.829473684 | 0.834654587 | 0.97875166 | 0.900977995 |
| Stepglm[both]+Enet[alpha=0.9] | 0.831578947 | 0.832772166 | 0.985391766 | 0.902676399 |
| Stepglm[backward]+Enet[alpha=0.9] | 0.829473684 | 0.830907055 | 0.985391766 | 0.901579587 |
| Stepglm[both]+Enet[alpha=0.1] | 0.831578947 | 0.832772166 | 0.985391766 | 0.902676399 |
| Stepglm[backward]+Enet[alpha=0.1] | 0.830526316 | 0.83258427 | 0.984063745 | 0.902008521 |
| Stepglm[both]+Enet[alpha=0.8] | 0.829473684 | 0.830907055 | 0.985391766 | 0.901579587 |
| Stepglm[backward]+Enet[alpha=0.8] | 0.829473684 | 0.830907055 | 0.985391766 | 0.901579587 |
| Stepglm[both]+Enet[alpha=0.2] | 0.831578947 | 0.83352081 | 0.984063745 | 0.902557856 |
| Stepglm[backward]+Enet[alpha=0.2] | 0.827368421 | 0.828316611 | 0.986719788 | 0.900606061 |
| Stepglm[both]+Lasso | 0.830526316 | 0.831838565 | 0.985391766 | 0.90212766 |
| Stepglm[backward]+Lasso | 0.829473684 | 0.830907055 | 0.985391766 | 0.901579587 |
| Stepglm[both]+Enet[alpha=0.6] | 0.833684211 | 0.833894501 | 0.986719788 | 0.903892944 |
| Stepglm[backward]+Enet[alpha=0.6] | 0.830526316 | 0.833333333 | 0.982735724 | 0.901889092 |
| glmBoost+GBM | 0.844210526 | 0.839506173 | 0.993359894 | 0.909975669 |
| Stepglm[both]+Enet[alpha=0.7] | 0.834736842 | 0.835585586 | 0.985391766 | 0.90432663 |
| Stepglm[backward]+Enet[alpha=0.7] | 0.832631579 | 0.833707865 | 0.985391766 | 0.903225806 |
| Lasso+Stepglm[backward] | 0.833684211 | 0.834645669 | 0.985391766 | 0.903775883 |
| Stepglm[both] | 0.830526316 | 0.834841629 | 0.980079681 | 0.901649359 |
| Stepglm[backward] | 0.831578947 | 0.835787089 | 0.980079681 | 0.902200489 |
| glmBoost+Stepglm[both] | 0.834736842 | 0.834080717 | 0.988047809 | 0.904559271 |
| glmBoost+Stepglm[backward] | 0.833684211 | 0.833146697 | 0.988047809 | 0.904009721 |
| Stepglm[both]+Enet[alpha=0.4] | 0.830526316 | 0.831838565 | 0.985391766 | 0.90212766 |
| Stepglm[backward]+Enet[alpha=0.4] | 0.829473684 | 0.832395951 | 0.982735724 | 0.901339829 |
| Stepglm[both]+Enet[alpha=0.3] | 0.831578947 | 0.832772166 | 0.985391766 | 0.902676399 |
| Stepglm[backward]+Enet[alpha=0.3] | 0.830526316 | 0.83258427 | 0.984063745 | 0.902008521 |
| Stepglm[both]+glmBoost | 0.831578947 | 0.830546265 | 0.98937583 | 0.903030303 |
| Stepglm[backward]+glmBoost | 0.834736842 | 0.834080717 | 0.988047809 | 0.904559271 |
| Stepglm[both]+Enet[alpha=0.5] | 0.834736842 | 0.836343115 | 0.984063745 | 0.904209884 |
| Stepglm[backward]+Enet[alpha=0.5] | 0.830526316 | 0.831096197 | 0.986719788 | 0.902246509 |
| Lasso+GBM | 0.846315789 | 0.8421646 | 0.992031873 | 0.91097561 |
| GBM | 0.847368421 | 0.842342342 | 0.993359894 | 0.911639244 |
| Stepglm[both]+SVM | 0.836842105 | 0.832962138 | 0.993359894 | 0.906117505 |
| Stepglm[backward]+SVM | 0.836842105 | 0.832962138 | 0.993359894 | 0.906117505 |
| Stepglm[both]+GBM | 0.850526316 | 0.842105263 | 0.998671979 | 0.913730255 |
| Stepglm[backward]+GBM | 0.850526316 | 0.842105263 | 0.998671979 | 0.913730255 |
| Stepglm[both]+RF | 0.835789474 | 0.829106946 | 0.998671979 | 0.906024096 |
| LDA | 0.850526316 | 0.857309942 | 0.973439575 | 0.911691542 |
| glmBoost+LDA | 0.862105263 | 0.867612293 | 0.974767596 | 0.918073796 |
| Stepglm[both]+LDA | 0.847368421 | 0.859338061 | 0.965471448 | 0.909318324 |
| Stepglm[backward]+LDA | 0.846315789 | 0.858323495 | 0.965471448 | 0.90875 |
| Lasso+LDA | 0.862105263 | 0.870238095 | 0.970783533 | 0.917765223 |
| Stepglm[backward]+RF | 0.845263158 | 0.837416481 | 0.998671979 | 0.910963053 |
| XGBoost | 0.844210526 | 0.843359818 | 0.986719788 | 0.909424725 |
| Lasso+XGBoost | 0.848421053 | 0.847206385 | 0.986719788 | 0.911656442 |
| glmBoost+XGBoost | 0.853684211 | 0.853686636 | 0.984063745 | 0.914250463 |
| Stepglm[both]+XGBoost | 0.855789474 | 0.85483871 | 0.985391766 | 0.915484269 |
| Stepglm[backward]+XGBoost | 0.852631579 | 0.851894374 | 0.985391766 | 0.913793103 |
| NaiveBayes | 0.831578947 | 0.835028249 | 0.981407703 | 0.902319902 |
| Lasso+NaiveBayes | 0.836842105 | 0.839002268 | 0.982735724 | 0.905198777 |
| glmBoost+NaiveBayes | 0.835789474 | 0.837288136 | 0.984063745 | 0.904761905 |
| Stepglm[both]+NaiveBayes | 0.824210526 | 0.831447964 | 0.976095618 | 0.897984117 |
| Stepglm[backward]+NaiveBayes | 0.822105263 | 0.829571106 | 0.976095618 | 0.896888347 |
